# Quantile function modeling with application to salinity tolerance analysis of plant data

**DOI:** 10.1186/s12870-019-2039-9

**Published:** 2019-11-28

**Authors:** Gaurav Agarwal, Stephanie Saade, Mohammad Shahid, Mark Tester, Ying Sun

**Affiliations:** 1 Computer, Electrical and Mathematical Science and Engineering Division, King Abdullah University of Science and Technology, Thuwal, 23955-6900 Saudi Arabia; 2 Biological and Environmental Science and Engineering Division, King Abdullah University of Science and Technology, Thuwal, 23955-6900 Saudi Arabia; 3 International Center for Biosaline Agriculture (ICBA), Dubai, United Arab Emirates

**Keywords:** Bivariate quantiles, Conditional quantiles, Joint estimation, Plant growth, Stress tolerance, Yields

## Abstract

**Background:**

In plant science, the study of salinity tolerance is crucial to improving plant growth and productivity under saline conditions. Since quantile regression is a more robust, comprehensive and flexible method of statistical analysis than the commonly used mean regression methods, we applied a set of quantile analysis methods to barley field data. We use univariate and bivariate quantile analysis methods to study the effect of plant traits on yield and salinity tolerance at different quantiles.

**Results:**

We evaluate the performance of barley accessions under fresh and saline water using quantile regression with covariates such as flowering time, ear number per plant, and grain number per ear. We identify the traits affecting the accessions with high yields, such as late flowering time has a negative impact on yield. Salinity tolerance indices evaluate plant performance under saline conditions relative to control conditions, so we identify the traits affecting the accessions with high values of indices using quantile regression. It was observed that an increase in ear number per plant and grain number per ear in saline conditions increases the salinity tolerance of plants. In the case of grain number per ear, the rate of increase being higher for plants with high yield than plants with average yield. Bivariate quantile analysis methods were used to link the salinity tolerance index with plant traits, and it was observed that the index remains stable for earlier flowering times but declines as the flowering time decreases.

**Conclusions:**

This analysis has revealed new dimensions of plant responses to salinity that could be relevant to salinity tolerance. Use of univariate quantile analyses for quantifying yield under both conditions facilitates the identification of traits affecting salinity tolerance and is more informative than mean regression. The bivariate quantile analyses allow linking plant traits to salinity tolerance index directly by predicting the joint distribution of yield and it also allows a nonlinear relationship between the yield and plant traits.

## Background

Soil salinity is a major abiotic stress that negatively impacts agriculture, as plants grown under saline conditions grow more slowly and have lower yields than plants grown under non-saline conditions [1]. Therefore, understanding mechanisms of salinity tolerance in plants is important to improve plant growth and productivity. Plants are able to maintain growth in saline conditions relative to non-saline conditions using a range of mechanisms, where a range of traits can contribute to this maintenance of growth and yield. Munns and Tester [2] suggested three main traits contributed to salinity tolerance: exclusion of toxic salts from the shoot, tolerance of toxic salts in the shoot that were not excluded from the shoot; and tolerance processes that were independent of shoot salt effects. These considerations have been developed further by Morton et al. [3] to include a wider range of other physiological traits, focusing in particular on the ability of plants to maintain processes in saline conditions relative to non-saline conditions. The technical approaches that can be taken to measure these traits is detailed in Negrao et al. [4].

To study salinity tolerance, a typical way is to define salinity tolerance indices, which measure the plant performance in saline conditions relative to non-saline conditions [5–7]. These indices are univariate and result in the reduction of the dimensions of data. As a consequence, a single index might not be sufficient to summarize the relationship between the indices and the covariates. In this paper, we apply a set of quantile analysis methods and demonstrate the necessity of these methods by studying the dependence of plant traits on salinity tolerance of barley accessions. A conventional statistics tool used to investigate the relationship between a response variable and covariates is the mean regression [8, 9]. Mean regression only provides an incomplete picture of the response distribution corresponding to the covariates, just as the mean does by providing an incomplete summary of a single distribution, and not accounting for extreme values in the data [10, 11].

Quantiles are the values that divide the entire distribution such that a given proportion of values, say *p*, lie below the *p*^th^ quantile, where *p*∈(0,1) [12]. For example, median is the 0.5^th^ quantile. The data can be divided into different quantiles, and we can check how the data is behaving for each quantile. On the other hand, mean provides a grand summary of the distribution by computing its average; hence losing information. Mean regression models the average of the distribution of the response variable for given covariates, assuming that the variables behave similarly at the upper and lower tails of the distribution as well as the mean. On the other hand, quantile regression models the entire distribution of the response, given the covariates, and provides a more comprehensive analysis of the effect of the predictors on the response [10, 13]. Quantile regressions are particularly valuable in applications where extremes are imperative, such as agricultural studies for which higher quantiles of yield are critical [14]. The regression that involves modeling the conditional mean of the response distribution might obscure the effect of a trait on the tails of the response, whereas quantile regression can reveal those effects. For instance, one particular trait may have a negligible effect on conditional means but may lower conditional 10^th^ percentiles sharply [15].

Quantile regression has drawn considerable research interest in recent years and is being applied in various fields. Quantile regression is becoming adapted in ecology and environmental sciences [16–18]. For instance, in some ecological applications, the approach of quantile regression was used to estimate the upper quantiles of the growth rates of marine phytoplanktons as a function of temperature [19] and to reveal the uncertainty in the relationship between an organism and its habitat at different quantile levels [20]. It has been used in biology to test the significance of dissolved oxygen concentration at the upper quantiles of body size of deep-sea organisms [21, 22]. Quantile regression has long been used in other disciplines, like business and economic analysis [23–26]. Methods based on quantile regression have been used in health and medicine and demonstrated how richer inferences could be drawn using quantile regression [27, 28]. In this paper, we are suggesting to extend the application of quantile regression techniques to the field of agriculture and salinity tolerance.

In case of a univariate distribution, the natural ordering of a variable is the order on real line $\mathbb {R}$. Hence, obtaining quantiles, in that case, is straightforward. However, for a bivariate distribution, there is no natural ordering of observations, and thus obtaining bivariate quantiles is statistically challenging [29–31], since we need to consider not only values but also directions. Kong and Mizera [32] proposed directional quantiles and directional quantile envelopes to characterize multivariate distribution. Using directional quantile envelopes, we propose here a bivariate quantile regression model to predict the behavior of the bivariate response variable jointly for given covariates.

To illustrate the methods we used, we perform a salinity tolerance analysis to evaluate the performance of barley accessions. We focus on the upper tails of the response distribution, as the accessions that are highly salt-tolerant and have a high yield in non-saline conditions are of primary interest. We perform a quantile regression analysis using plant agronomic traits, and a salinity tolerance index to identify the traits that affect the accessions with high indices. We also propose a flexible approach to identify accessions with high salinity tolerance along with high yield using conditional and marginal quantiles. We predict the bivariate distribution of plant yield under two different conditions (non-saline and saline), for a given plant trait, and hence, directly associate salinity tolerance indices with the plant traits to get a detailed analytic understanding of the effects of plant traits on salinity tolerance. The dataset presented in this paper is used to provide an example of how the quantile analysis methods can be applied to the field of agriculture and salinity tolerance.

## Results

### Behaviour of traits in non-saline and saline conditions

**Fig. 1:**
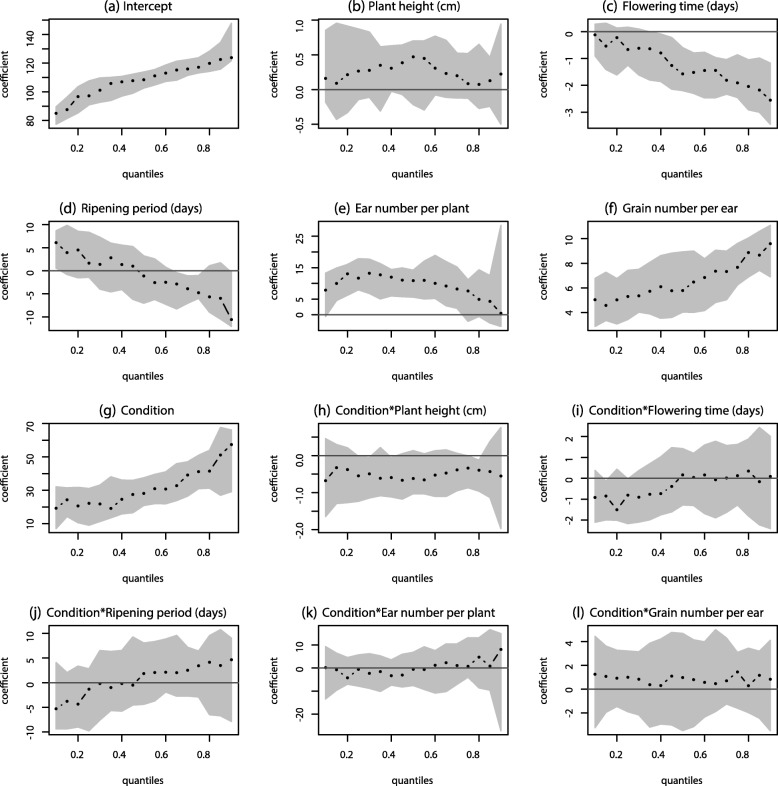
Relationship between plant traits and yield explained through quantile regression modeling: Each panel represents the estimated value of regression coefficient in black dots and the estimated 95% confidence interval in grey area as a function of quantile level for respective covariate in the model with yield as response, obtained by fitting a multiple quantile regression model (n=808). The horizontal black lines represent the zero value of the estimated coefficients. The variable condition is a dummy variable and was coded 1 for non-saline conditions and 0 for saline conditions. a*b represents the interaction between covariates a and b. **a** Intercept, **b** Plant height (cm), **c** Flowering time (days), **d** Ripening period (days), **e** Ear number per plant, **f** Grain number per ear, **g** Condition, **h** Condition*Plant height (cm), **i** Condition*Flowering time (days), **j** Condition*Ripening period (days), **k** Condition*Ear number per plant, **l** Condition*Grain number per ear

In this section, we perform a univariate quantile regression under both non-saline and saline conditions using a common model, for different quantile levels, to observe the behavior of the traits on the complete distribution of the response yield. The plot of the results of the fitted quantile regression model is shown in Fig. 1. Since the categorical variable condition was coded as 0 for saline conditions and 1 for non-saline conditions, the individual estimated effects represent the results for saline conditions, and the interaction terms represent the difference between the estimated effects of each covariate for the accessions with non-saline and saline conditions. We can observe that condition is positively significant for all quantile levels since the estimated confidence interval does not include the horizontal line for zero value of the estimated coefficent (Fig. 1g), which means that for an average value of plant traits, the yield in non-saline conditions is significantly greater than the yield in saline conditions. The change in slope at higher quantiles means that the difference is higher for accessions with higher yields.

Besides, the difference in yield under the two conditions can be attributed to the height of accessions, since the interaction term of plant height is significant at some quantiles (Fig. 1h). As the plant height increases, the yield in non-saline conditions decreases, while plant height does not significantly affect the yield in saline conditions. Ripening period positively affects yield in saline conditions for low and mid quantiles but negatively affects yield for higher quantiles (Fig. 1d).

We also found that the flowering time seems to have a negative impact on yield in saline conditions, for accessions with mid-level and high yield (Fig. 1c). This effect is more substantial for accessions with a high yield than mid-level yield which can be seen from the change in slope, while this observation is not significant for accessions with a low yield. Based on the differences, the negative effects of flowering time at mid and high quantiles are similar in non-saline conditions but has stronger negative effects on yield at lower quantiles compared to saline conditions (Fig. 1i). Ear number per plant (Fig. 1e), and grain number per ear (Fig. 1f) have a significant positive impact on yield in saline conditions. The impact of grain number per ear on yield under saline conditions is more substantial for accessions with high yield. The interaction terms for variables except for plant height and flowering time, are not significant, so there is no significant difference in the estimated effects of ripening period (Fig. 1j), ear number per plant (Fig. 1k), and grain number per ear (Fig. 1l) on yield between non-saline and saline conditions.

The results of a similar framework using mean regression for the same model is shown in Table 1. From these results, we can merely comment that, on average, accessions with late flowering time have a lower yield. Quantile regression reveals that this effect is not significant for accessions with low yield (lower quantiles). Also, a later flowering time affects accessions with high yield more than it does for the accessions with average yield.

**Table 1:** Results of mean regression between plant traits and yield

Traits	Coefficient value	Standard error	*p*-value
Intercept	109.9237	3.5151	2e-16 ^∗^
Plant height (cm)	0.3526	0.2329	0.130530
Flowering time (days)	-1.1282	0.3195	0.000438 ^∗^
Ripening period (days)	-0.8952	2.5387	0.724464
Ear number per plant	10.3050	3.2122	0.001390 ^∗^
Grain number per ear	6.7732	0.7903	2e-16 ^∗^
Condition	27.7525	4.2314	9.77e-11 ^∗^
Condition × Plant height (cm)	-0.6567	0.2811	0.019716 ^∗^
Condition × Flowering time (days)	-0.2733	0.4232	0.518699
Condition × Ripening period (days)	1.2312	2.9102	0.672357
Condition × Ear number per plant	-0.5443	3.6711	0.882178
Condition × Grain number per ear	0.9860	1.0241	0.335920

The three columns represent the estimated coefficient value, standard error and the *p*-value for the respective covariate obtained by fitting a multiple mean regression model with sample size 808. The significant *p*-values are marked with a ‘*’, for a significance level of 0.05. The variable condition is a dummy variable and was coded 1 for non-saline conditions and 0 for saline conditions. a × b represents the interaction between a and b

Mean regression also shows that, with an increase in grain number per ear, on average, the yield in saline conditions increases by 6.7 g/ m^2^. While with quantile regression, we can observe for accessions with high yield, the increase is nearly 10 g/ m^2^. Therefore, mean regression provides limited opportunity for studying the accessions with extreme yields which are of utmost agronomic importance, while quantile regression allows us to fine-tune the relationship between a trait and yield at different quantiles.

### Traits affecting salinity tolerance indices

**Fig. 2:**
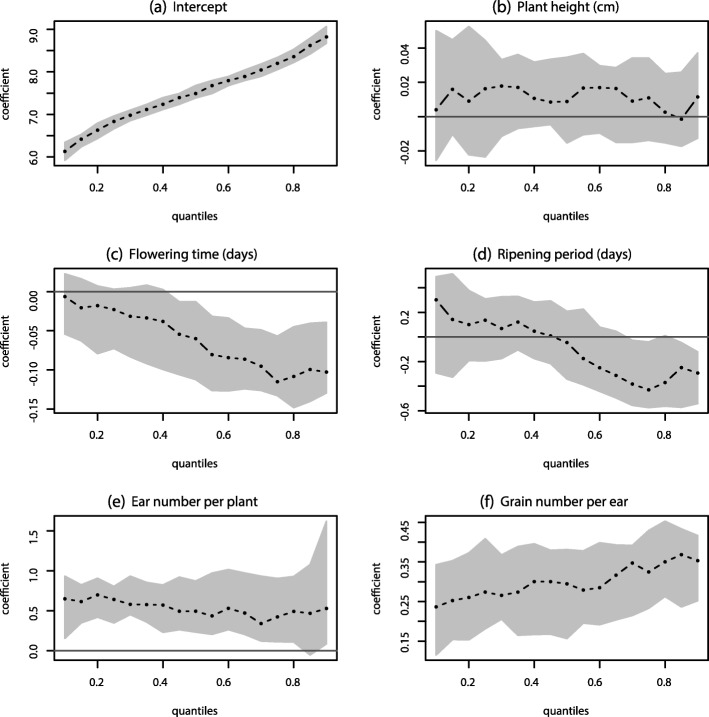
Relationship between plant traits in saline conditions and SWP index explained through quantile regression modeling: Each panel represents the estimated value of regression coefficient in black dots and the estimated 95% confidence interval in grey area as function of quantile level for each covariate in the model with SWP index as response, obtained by fitting a multiple quantile regression model (*n*=404). The horizontal black lines represent the zero value of the estimated coefficients. **a** Intercept, **b** Plant height (cm), **c** Flowering time (days), **d** Ripening period (days), **e** Ear number per plant, **f** Grain number per ear

To study the characteristics of a specific set of response variables that may be important in the context of salinity tolerance, we investigate the tail behavior of the response using quantile regression. One of our goals is to examine the accessions with a high salinity tolerance index. Several salinity tolerance indices have been previously proposed to identify stress tolerant and high-yielding accessions [5, 6, 33]. Saade et al. (2016) [33] shows how SWP is better than other salinity tolerance indices (S/C and STI) in selecting accessions that are salt tolerant and have high yield. Using our dataset, we compute SWP using yield under saline and non-saline conditions for each accession and conduct a trait analysis to assess the significance of traits affecting the salinity tolerance. Here, we consider the effect of the traits under saline conditions on SWP, which is considered as the response. We perform a quantile regression on SWP using plant traits under saline conditions as covariates (n= 404); we then check for the significance of plant traits that affect the salinity tolerance of plants. The results for the quantile regression model on SWP are shown in Fig. 2.

We observed that late flowering time has a negative impact on salinity tolerance, with the accessions with high SWP being affected the most, and it has no significant impact on accessions with low SWP (Fig. 2c). This could be explained by the fact that plants that flower later are more exposed to the heat and plants with low SWP are already struggling with the salt stress. Quantile regression helped us observed that the ripening period is not significant for accessions with median SWP, but it is negatively significant for accessions with high SWP (Fig. 2d). It was also observed that ear number per plant (Fig. 2e) and grain number per ear (Fig. 2f) have a significant positive impact on salinity tolerance index SWP. The effect of grain number per ear is more substantial for accessions with high SWP than with median SWP.

Since SWP is used to differentiate the top-performing accessions from the other accessions based on the order of their values, accessions with high values of the index are of more practical importance. Using the quantile analyses, we study the effects of plant traits on accessions with high salinity tolerance, whereas, from the results of mean regression, we can only comment on accessions with average salinity tolerance, and therefore do not have any information on accessions with different ranges of salinity tolerance. The results of the mean regression for salinity tolerance indices are shown in Table 2. It shows that, on average, the ripening period does not have a significant effect on SWP, but quantile regression revealed that the ripening period is significant for the high quantiles of SWP.

**Table 2:** Results of mean regression between plant traits in saline conditions and SWP index

Traits in saline conditions	Coefficient value	Standard error	*p*-value
Intercept	7.506834	0.054184	2e-16 ^∗^
Plant height (cm)	0.014669	0.009262	0.114
Flowering time (days)	-0.050227	0.012705	9.12e-05 ^∗^
Ripening period (days)	-0.065254	0.100945	0.518
Ear number per plant	0.588800	0.127725	5.44e-06 ^∗^
Grain number per ear	0.291359	0.031424	2e-16 ^∗^

The three columns represent the estimated coefficient value, standard error and the *p*-value for the respective covariate in the model with SWP as the response, obtained by fitting a multiple mean regression model with sample size 404. The significant *p*-values are marked with a ‘*’, for a significance level of 0.05

### High salt tolerant and high yielding accessions

**Fig. 3:**
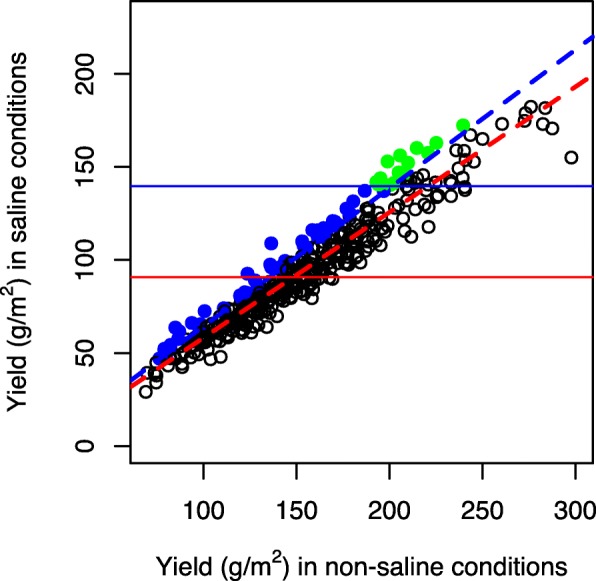
High salt tolerant and high yielding accessions obtained using conditional and marginal quantiles: The top-performing accessions with respect to high tolerance and yield are identified with filled green dots. The dashed blue and red lines represent the fitted lines of quantile regression for quantile level 0.85 and 0.5 respectively, and the solid blue and red lines are marginal quantiles of level 0.9 and 0.5 respectively. The accessions above conditional quantile level 0.85, but below 0.9 marginal quantile levels are denoted by blue dots, and represent accessions with high salt tolerance but not necessarily with high yield

Saade et al. [33] showed how SWP outperforms STI in terms of selecting salt-tolerant accessions and how it chooses accessions with higher marginal yield than those chosen by S/C. Here, we propose a flexible approach to classify the observations using conditional and marginal quantiles. The conditional and marginal quantile levels can be chosen by practitioners according to their interest as a trade-off between high salt tolerance of accession and high yield. The observations are classified using the intersection of the fitted univariate quantile regression line and marginal quantile line. We consider the distribution of yield under saline and non-saline conditions to illustrate the use of this method. Here, we are interested in accessions with a high stress tolerance together with high yield under non-saline conditions. Accessions lying above the fitted conditional quantile line, for yield under saline conditions conditioned on non-saline conditions, are salt tolerant, while those lying above the marginal quantile of yield under saline conditions have a high yield. We take the intersection of both conditions and obtain the top-performing accessions. This method was applied to yield under saline and non-saline conditions of barley. The best performing accessions in terms of both salt tolerance and high yield are identified with green circles lying above the 85^th^ conditional and 90^th^ marginal quantiles (Fig. 3).

### Bivariate prediction of yield for given traits

The salinity tolerance indices depend on yield from both saline and non-saline conditions, so to link an index to a plant trait, we need to model the joint distribution of yield for that plant trait. We applied the method of directional quantiles [32] to estimate the empirical distribution of our bivariate data, non-parametrically. Using the directional quantile envelopes [32], we defined a way to predict the bivariate vector of yield for a given covariate. We linked the covariates of the yield under saline conditions with the bivariate data. We predicted these envelopes for a given value of the plant trait. Three *p*^th^ directional quantile envelopes (also known as depth contours) were predicted, corresponding to *p*=*p*^∗^,0.25,0.1; they were called the median, inner and outer envelope respectively, with *p*^∗^ being the highest quantile value obtained for a non-empty quantile envelope in *p*∈(0,1/2], which has the highest depth. Since the observation corresponding to the largest depth value in the data cloud is the deepest value, it is referred to as, multivariate median [34], we named the envelope corresponding to the highest depth value obtained, the median envelope.

**Fig. 4:**
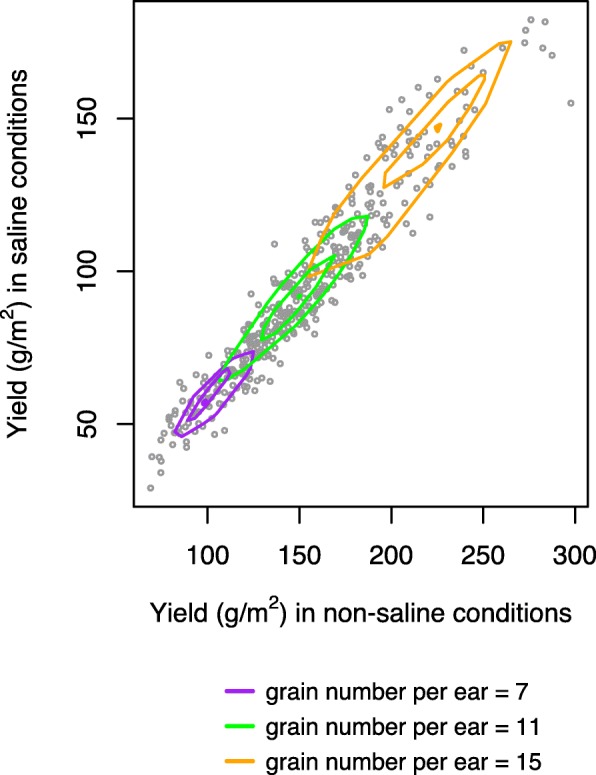
Bivariate regression to predict the joint distribution of yield under non-saline and saline conditions for a given plant trait: The predicted quantile envelopes for three values of grain number per ear: 7, 11, 15 for *p*=0.1, 0.25, *p*^∗^ forming the outer, inner and median envelope respectively. The different colors indicate different values of grain number per ear

Figure 4 shows the predicted envelopes for three values of grain number per ear: 7, 11, 15. These values are chosen from lower, median and upper quantiles of the trait distribution so that the envelopes do not over plot. These envelopes demonstrate the dependence of increasing grain number per ear for *p*=0.1, *p*=0.25, *p*=*p*^∗^, forming the outer envelope, inner envelope, and the median envelope respectively. The directional quantile envelopes move upward along the data cloud, showing the dependence on increasing the covariate grain number per ear.

**Fig. 5:**
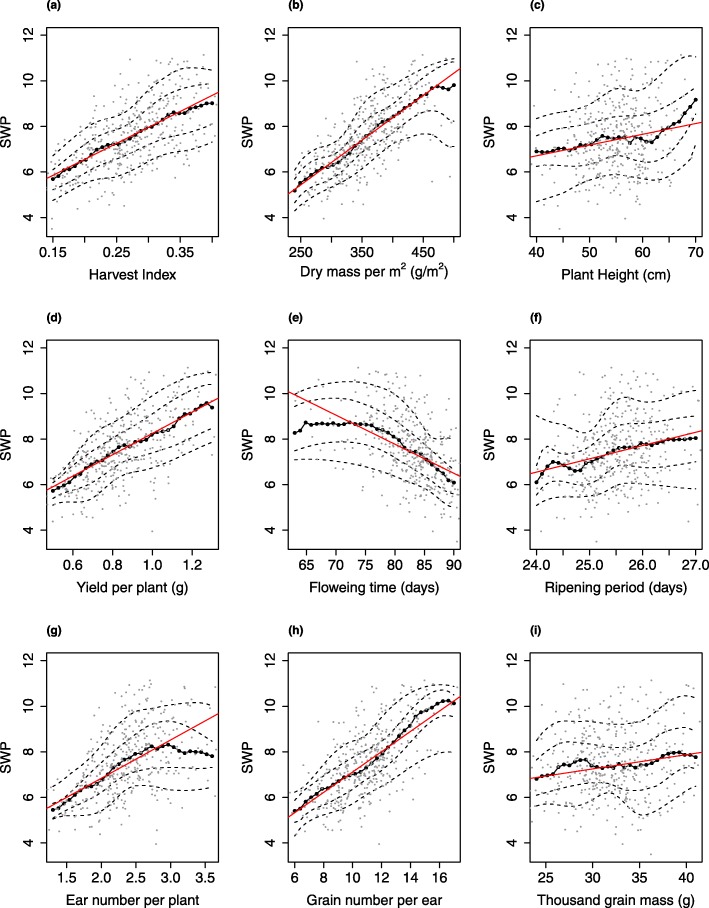
Relationship between plant traits and stress-weighted performance index (SWP) predicted using bivariate regression: Each of the nine panels represents a scatter plot between a plant trait and SWP in grey dots. The solid black line represents the predicted values of the index using bivariate regression, the dashed black lines are the lower and upper confidence bands, formed using inner and outer envelopes respectively, and superimposed is the median regression line in red

For a given value of grain number per ear, the bivariate distribution of yield was estimated from the bivariate median. SWP is then estimated from the bivariate regression estimates of yield in saline and non-saline conditions. Using the bivariate regression estimates, we can compute the estimate of any stress tolerance index for a given plant trait since they are functions of yield in both conditions. We obtain a comprehensive view of how the salinity tolerance index varied for a given plant trait. Figure 5 demonstrates the effect of each plant trait, taken one at a time, on the salinity tolerance index SWP.

The results of the bivariate quantile regression were then compared with those obtained using a standard univariate quantile regression method. We observed an apparent increase in the value of the index as the grain number per ear (Fig. 5h), ear number per plant (Fig. 5g) and dry mass per m^2^ (Fig. 5b) increases. However, the increase was not always linear, and the slope varied with the range of values of covariates. The stress-weighted performance index (SWP) increased linearly as the ear number per plant increased to 3; it then stopped increasing, even with the further rise in ear number per plant (Fig. 5g). A possible explanation is that the plant can still cope with the salt stress while producing seeds, but up to 3 ear number per plant, after which, producing more seeds comes at the expense of salinity tolerance. SWP remained approximately constant for small values of flowering time, and then it decreased linearly as the flowering time increased (Fig. 5e). A possible explanation is that the yield of late-flowering plants grown under saline conditions is also affected by heat as the season progresses.

## Discussion

The study of salinity tolerance is crucial to improve crop yield in salt-affected areas. We provide methods to study the effect of plant traits on salinity tolerance. We show that the quantile analysis methods are advantageous over mean regression methods for studying the relationship between covariates and the entire distribution of response by performing a salinity tolerance analysis. Using quantile analyses, we study traits affecting plants with extreme yields and high salinity tolerance indices.

The univariate quantile analysis is a simple approach that yet gives a thorough visualization of how the plant traits affect the complete distribution of yield for both non-saline and saline conditions and also the difference in the estimated effects between the two conditions. A limitation of this study is that the standard errors of the estimates were high, due to small sample sizes and some of the variables might be nonsignificant due to high uncertainty of the estimated values.

We propose bivariate quantile analysis methods to study the effect of plant traits on salinity tolerance indices. The added value of using bivariate quantile regression is that it provided the ability to predict the bivariate response for a given value of covariate, whereas the traditional method of quantile regression reduced the response to univariate, by taking the ratio of the yield under saline conditions to the square root of yield under non-saline conditions. As previously mentioned, computing a stress index reduces dimensionality to one; hence, we lose information. In the method we used, we do not need to compute the index before making the prediction; we can find the relationship of the plant traits with any salinity tolerance index, once we have predicted the bivariate distribution of yield. Another advantage of using this bivariate quantile regression is that it allows nonlinearities in response by using cubic splines on the covariate. Also, since the yield in two conditions is related, it is favorable to model the joint distribution of yield for a given plant trait instead of modeling merely a univariate function of the two components of yield. With bivariate quantile regression, we have the estimates yield in saline and non-saline conditions for a given plant trait, but with conventional univariate regression, we cannot recover the individual estimates of yield. Although the model described for bivariate quantile regression studies the effect of only a single covariate on the response at a time, it could be extended to study multiple covariates by including spline functions for multiple covariates in the model.

## Conclusions

From the different quantile analysis approaches we used in this paper, we made new observations and found out information that could not be previously obtained from analyses such as those presented in Saade et al. (2016) [33]. From quantile analyses, using yield and plant traits under saline and non-saline conditions, we observed the effects of plant traits on yield. We observed that a late flowering time has a negative impact on yield in saline conditions, for accessions with high yield. From quantile analyses, using SWP index, we noticed that an increase in ear number per plant and grain number per ear increase the salinity tolerance index and in case of grain number per ear the rate of increase is larger for accessions with high yield. On the contrary, a late flowering time decreases the salinity tolerance index for accessions with high yield. The use of conditional and marginal quantiles provides a flexible approach for selecting high yielding and high salinity tolerant accessions. From bivariate quantile analysis methods, we observed that SWP remains stable for earlier flowering times and then starts declining as the flowering time increases. SWP increases with an increase in the ear number per plant, and then stabilizes for higher values without any further increase, while it continuously increases for grain number per ear. These observations are biologically relevant and may impact on our understanding of mechanisms of tolerance to salinity.

## Methods

### Plant material

The plant material consisted of 404 barley accessions from a barley association mapping (AM) population provided by Prof. Robbie Waugh from the James Hutton Institute, United Kingdom. All accessions were 2-row spring barley cultivars.

### Field trial

Plants from the AM population were grown at the International Center for Biosaline Agriculture (ICBA, Dubai), over a year, from 2013 to 2014. Plots were irrigated with fresh (1 dS/m; referred to as ‘non-saline’) and saline water (17 dS/m; referred to as ‘saline’). An augmented design was used where salt tolerant check lines (116/2A, 58/1A, and CM72) were added every seven plots on average. Detailed descriptions of the field design and practice are provided by Saade orton (2016), who grew and studied another population, HEB-25 [33]. The following plant traits were recorded under both conditions: flowering time (days), ripening period (days), plant height (cm), ear number per plant, grain number per ear, thousand grain mass (g), dry mass per m^2^ (g/ m^2^), grain mass per m^2^ (g/ m^2^, referred to as yield), and harvest index. A detailed description of each trait and how it was measured is provided in Saade et al. (2016) [33]. The experiment that generated the raw phenotypic data used in this paper was originally designed for an association mapping analysis of salinity tolerance in barley.

### Univariate quantile analyses

As defined in the book “Quantile Regression” by Roger Koenker [35], for a given real-valued random variable *Y* with a distribution function *F*, the *p*^th^ quantile is given by

$$Q(p) = F^{-1}(p)=\inf \{y : F(y) \ge p \} \quad \text{for} \quad 0< p<1. $$

If we denote the *p*^th^ conditional quantile function as *Q*_*y*_(*p*∣***x***)=***x***^*T*^**β**(*p*), the optimization problem of quantile regression can be formulated as

$$\min_{\beta \in \mathbb{R}^{p}} \sum_{i=1}^{n}\rho_{p}\left(y_{i}-\boldsymbol{x}_{i}^{T}\mathbf{\beta}\right), $$

where $\rho _{p}(u)=u(p-\mathbbm {1}{(u<0)})$ is the loss function, and $\mathbbm {1}(\cdot)$ is an indicator function. The *y*_*i*_’s represent the realizations of the response variable; ***x*** is the design matrix with the first column as the unit vector, and the rest of the columns represent the values of the covariates; **β** is the vector of regression coefficients. Regression coefficients of a quantile regression model are estimated by minimizing the loss function *ρ*_*p*_(*u*). We include the saline and non-saline groups in a common model by including a categorical covariate for that condition classification and adding its interactions with all the other covariates. This allows the analysis to not only test and estimate the effects of covariates for the saline and non-saline groups separately, but also provide the possibility of testing and estimating the differences between the estimated effects of each covariate for the non-saline and saline groups. The categorical variable for the classification of non-saline and saline conditions was coded as a dummy variable. The model used for univariate quantile regression is given by

$$y_{i} = \beta_{0} + \sum_{j=1}^{p} \beta_{j} {x_{ij}} + \alpha_{0}D_{i} + \sum_{j=1}^{p} \alpha_{j} D_{i} {x_{ij}} + \epsilon_{i}, \quad i=1,\dots,n, $$

where *y* is the response, *β*_0_, *α*_0_, *β*_*j*_ and *α*_*j*_, *j*=1,…,*p* are regression coefficients, *x*_*j*_, *j*=1,…,*p* are covariates, *D* is a dummy variable: *D*_*i*_=1 if *i*∈ non-saline group and *D*_*i*_=0 if *i*∈ saline group and *ε* is random error. Here, *Dx*_*j*_ denotes the interaction terms.

We eliminate the variables harvest index, thousand grain mass (g), and dry mass per m^2^ from our multiple regression model as they are partial expressions of yield, the response variable, so it might not be useful to study their effects, and they could cause the problem of multicollinearity. After dropping these variables, the variance inflation factors (VIFs) for all the plant traits was less than 2.5, so we consider all other plant traits as covariates in the model. The sample size for the model was *n*=808 (404 for saline and 404 for non-saline condition). We do not scale the covariates to unit variance as we notice no advantage gained by scaling and indeed, estimated effects are far more interpretable in their original units. We center the covariates just so that their mean is 0 and thus the intercept represents the response (yield) at the mean of all predictors. The model is fit using the rq() function of the quantreg package in R for quantile levels ranging from 0.1 to 0.9. Although we are interested in studying the accessions with high yield, we investigate the model for the whole range of quantiles, which allows us to check for the stability in the coefficient value and examine the change in slope as we move from lower quantile to upper quantiles. After fitting the quantile regression model, we plot the estimated values of coefficients and the estimated 95% confidence intervals of the plant traits as a function of the quantile level to examine the relationship between the plant trait and different quantiles of yield. The upper and lower bounds for the estimated quantile regression coefficients are calculated using the rankscore test inversion [36]. This method is suitable in case of small sample sizes (less than 1000). The assumption of independent and identically distributed errors is also relaxed [37]. The test of significance is determined using the confidence intervals produced by rank inversion method. If the estimated confidence interval around the observed effect includes 0, then the effect is not statistically significant.

By plotting the estimated regression coefficients along with the estimated confidence interval against the quantile level, we were able to give a complete picture of the relationship between traits and response distribution in both the non-saline and saline conditions separately and also on the differences between the two conditions.

We also performed a quantile regression analysis on salinity tolerance index SWP [33] (stress-weighted performance) for different quantile levels where the upper tails of response distribution were of principal interest. SWP is defined as

$$\text{SWP} = \frac{y_{\mathrm{s}}}{\sqrt{y_{\mathrm{c}}}}, $$

where *y*_s_ denotes the yield under saline conditions and *y*_c_ yield under non-saline conditions. The salinity tolerance index SWP was set as the response, and the plant traits from saline conditions were set as the covariates and were centered to mean 0. The model for univariate quantile regression for salinity tolerance index is given by

$$y_{i} = \beta_{0} + \sum_{j=1}^{p} \beta_{j} {x_{ij}} +\epsilon_{i}, \quad i =1,\dots,n, $$

where *y* is the response, *β*_0_, *β*_*j*_, *j*=1,…,*p* are regression coefficients, *x*_*j*_, *j*=1,…,*p* are covariates and *ε* is the random error. The standard errors were computed using the rankscore inversion test without the assumption of independent and identically distributed errors.

To identify high salt tolerant and high yielding accessions, we make use of conditional and marginal quantiles. We regress yield under non-saline conditions on yield under saline conditions using the model

$$y_{i} = \alpha x_{i} + \epsilon_{i}, \quad i=1,\dots,n, $$

where *y* is the response, *α* is the regression coefficient, *x* is the covariate and *ε* is random error. We fit a univariate quantile regression model to obtain the conditional quantile function *Q*_*y*∣*x*_(*p*_1_) for quantile level *p*_1_. We also obtain the marginal quantile function *Q*_*y*_(*p*_2_) for quantile level *p*_2_. The accession lying above the fitted conditional quantile line, i.e., the accessions with positive residuals are highly salt tolerant, while the accessions falling above the marginal quantile of *y*, will have a high yield in saline conditions, for chosen quantile levels *p*_1_ and *p*_2_. By taking the intersection of the two methods, we can identify highly salt tolerant and high yielding accessions.

### Web application for univariate quantile analyses

The method of univariate quantile regression analysis was implemented in a broader framework, in an open-source online application called MVApp [38]. The application was built using the Shiny framework of R. This method is available online at http://mvapp.kaust.edu.sa/MVApp/ and is freely and easily accessible. Users can upload their data on the application and choose their response, covariates, treatment and how they want to subset their data. The results of the analysis can be downloaded as a summary table and as plots.

### Bivariate quantile analyses

The goal of bivariate quantile analysis method is to predict the bivariate response, for a given covariate, by predicting directional quantile envelopes for the bivariate distribution. The notion of directional quantile envelope was proposed by Kong and Mizera [32] in 2012. In their approach, they project the bivariate distribution to univariate distribution along a direction ***s***, and obtain the quantiles of the projected distribution, calling them directional quantiles. Consider a normalized direction ***s***, on the unit circle $\mathcal {S}$, the *p*^th^ directional quantile of the random vector ***Y***, in direction ***s***, is defined by

$$Q(p,\boldsymbol{s})=\text{inf}\{\boldsymbol{y} : F(\boldsymbol{s}^{T}\boldsymbol{y})\ge p\}. $$

For *p*∈(0,1/2], the *p*^th^ directional quantile line is given by the equation ***s***^*T*^***y***=*Q*(*p*,***s***) which indicates how directional quantiles divide the data. The *p*^th^ directional quantile envelope produced by *Q*(*p*,***s***) is defined as the intersection,

$$D(p)= \bigcap_{\boldsymbol{s} \in \mathcal{S}} H(\boldsymbol{s},Q(p,\boldsymbol{s})), $$

where *H*(***s***,*q*)={***y***:***s***^*T*^***y***≥*q*} is the supporting halfspace. These envelopes are closely related to the Tukey depth contours proposed by Tukey in 1975 [34]. They are essentially Tukey depth level sets. The Tukey depth contours completely characterize the empirical distribution, for any multivariate dataset [39].

The directional quantile envelopes for bivariate data are non-empty for *p*≤1/3, because of a result known as the centerpoint theorem [40]. The points corresponding to the highest depth are the deepest [34, 41]. We obtain the highest value of *p*∈(0.33,0.5) for which the envelope is non-empty (for every case) and denote it by *p*^∗^; we call the envelope corresponding to *p*^∗^ as the median envelope since the envelope corresponding to the highest *p* will have the highest depth. We take the average of the vertices of the median envelope to obtain the bivariate median. We then choose two values, 0.1 and 0.25, and call the envelopes corresponding to these values the outer and inner envelopes, respectively.

To construct the *p*^th^ directional quantile envelopes for a given covariate, we need to obtain the *p*^th^ directional quantile for the given value of covariate in a subset of all the directions along a unit circle. For each direction ***s***, we model the projected distribution ***s***^*T*^***y*** using a cubic spline function of the given plant trait. Let $y_{s_{i}} = \boldsymbol {s}^{T} \boldsymbol {y_{i}}, i=1,\dots,n $, we fit the following quantile regression model for quantile *p*

$$\begin{array}{*{20}l} y_{s_{i}} &= {\beta_{0}} +{\beta_{1}}x_{i}+{\beta_{2}}x_{i}^{2}+ {\beta_{3}}x_{i}^{3} + \sum_{j=1}^{K} {\delta_{j}}(x_{i}-k_{j})^{3}_{+} + \epsilon_{i}, \\ i&=1,\dots,n, \end{array} $$

where *y* is the response, *x* is the covariate, *β*_0_, *β*_1_, *β*_2_, *β*_3_, and $\{\delta _{j}\}_{j=1}^{K}$ are regression coefficients, and $\{k_{j}\}_{j=1}^{K}$ are prespecified set of knots. The number of knots were fixed to 3 and the knots are typically chosen as suitable quantile of *x*. From the fitted model, we obtain the *p*^th^ directional quantile for a given value of covariate *x*, in direction ***s***, denoted by $\hat {Q}(p,\boldsymbol {s})$. Then the predicted *p*^th^ directional quantile envelope for a given value of covariate *x*, produced by $\hat {Q}(p,\boldsymbol {s})$ is defined as the intersection,

$$\hat{D}(p)= \bigcap_{\boldsymbol{s} \in \mathcal{S}} H(\boldsymbol{s},\hat{Q}(p,\boldsymbol{s})). $$

Hence, we predict the bivariate distribution of yield by predicting the bivariate median for a given covariate. The salinity tolerance indices are functions of yield under both conditions. So the predicted estimates of yield under both conditions from bivariate quantile regression were used to compute the salinity tolerance index SWP. After obtaining the estimates of yield in non-saline and saline conditions, we estimated SWP as

$$\hat{\text{SWP}} = \frac{\hat{y}_{\mathrm{s}}}{\sqrt{\hat{y}_{\mathrm{c}}}}, $$

where $(\hat {y}_{\mathrm {s}}, \hat {y}_{\mathrm {c}})$ are bivariate quantile regression estimates of yield under saline and non-saline conditions. Hence, for a given plant trait, we obtain SWP, which is capable of identifying top-performing accessions in terms of high yield and high salinity tolerance [33], together with its upper and lower bounds obtained from the predicted outer and inner envelopes.

## Data Availability

The dataset analysed during the current study is available in the Open Science Framework repository, (https://osf.io/wzhe7/).
